# Web-Based Antismoking Advertising to Promote Smoking Cessation: A Randomized Controlled Trial

**DOI:** 10.2196/jmir.6563

**Published:** 2016-11-21

**Authors:** Elad Yom-Tov, Peter Muennig, Abdulrahman M El-Sayed

**Affiliations:** ^1^ Microsoft Research Herzeliya Israel; ^2^ Columbia University New York, NY United States; ^3^ Detroit Health Department Detroit, MI United States

**Keywords:** smoking cessation, online advertising, computational advertising

## Abstract

**Background:**

Although hundreds of millions of dollars are spent each year on public health advertising, the advertisement content, design, and placement are usually developed by intuition rather than research.

**Objective:**

The objective of our study was to develop a methodology for testing Web-based advertisements to promote smoking cessation.

**Methods:**

We developed 10 advertisements that varied by their content (those that empower viewers to quit, help viewers to quit, or discuss the effects of smoking). We then conducted a series of Web-based randomized controlled trials that explored the effects of exposing users of Microsoft’s Bing search engine to antismoking advertisements that differed by content, placement, or other characteristics. Finally, we followed users to explore whether they conducted subsequent searches for smoking cessation products or services.

**Results:**

The advertisements were shown 710,106 times and clicked on 1167 times. In general, empowering advertisements had the greatest impact (hazard ratio [HR] 2.6, standard error [SE] 0.09 relative to nonempowering advertisements), but we observed significant variations by gender. For instance, we found that men exposed to smoking cessation advertisements were less likely than women to subsequently conduct smoking cessation searches (HR 0.2, SE 0.07), but that this likelihood increased 3.5 times in men exposed to advertisements containing empowering content. Women were more influenced by advertisements that emphasized the health effects of smoking. We also found that appearing at the top right of the page (HR 2.1, SE 0.07) or at the bottom rather than the top of a list (HR 1.1, SE 0.02) can improve smoking cessation advertisements’ effectiveness in prompting future searches related to smoking cessation.

**Conclusions:**

Advertising should be targeted to different demographic groups in ways that are not always intuitive. Our study provides a method for testing the effectiveness of Web-based antismoking advertisements and demonstrates the importance of advertisements that are tailored according to specific demographics.

## Introduction

In the United States alone, tens of millions of public health dollars are spent annually on advertisement campaigns in the belief that it is possible to change smoking behaviors [1]. For example, the Centers for Disease Control and Prevention’s Tips From Former Smokers campaign, which ran in 2013, cost roughly US $48 million [2]. However, it is difficult to know which advertisements are effective and which are not. Research into the effectiveness of advertisements often relies on weaker scientific evaluation methods, such as before-and-after designs [3] or designs that measure very long-term population-level effects.

As public health advertisements move onto the Web, however, it has become possible to conduct randomized controlled trials (RCTs)—the gold standard in research design—of smoking advertisement content and characteristics. This can be done by exposing users to advertisements, and then following these users on the Web to measure the influence of these advertisements on the users’ health risk behaviors as observed in their searches [4-6]. For example, investigators can measure subsequent searches, key words used in online posts or emails, shopping behavior [7], or exercise behavior (eg, as measured by global positioning systems), providing a robust picture of the outcomes associated with Web-based advertisements. This can be done quickly and at a fraction of the cost of a real-world trial.

The private sector has long used such inexpensive experiments to test products [4-6]. For instance, Google and Microsoft often test the impact of website design or user interfaces by randomly exposing participants to different design concepts and then observing participants’ responses. For example, the *New York Times* tested the effects of various font types on readers’ perceptions of the validity of the same block of text by randomly changing the font and then surveying the readers about their perceptions of the text [8]. Likewise, researchers have experimentally manipulated the number of positive or negative posts that users saw on Facebook, and then examined the emotional content of users’ subsequent posts [9].

Search engine queries are known to reflect real-world behaviors [10]. As such, researchers have used search engine queries to infer behaviors in the real world, both health related and otherwise [11], and their effect on future health outcomes. The use of Web-based advertising to measure health outcomes was pioneered by Eysenbach [12]. Web-based advertisements have also been used to measure sentiment to behaviors on the Internet [13] and to increase consumer demand for smoking cessation interventions [14]. However, the use of Web-based advertising to induce a behavioral change and its measure through search engine queries has not, to our knowledge, been attempted thus far.

In theory, a mix of Web-based advertisements can be developed and targeted to specific populations. Users can then be randomly shown advertisements within this mix to determine whether they had the intended effect on the intended audience. Using these data, it should be possible to continuously refine advertisements such that they can have ever-increasing effectiveness in changing individual behavior.

Therefore, the goal of this study was to take the first step toward proving this concept by developing a methodology for testing Web-based advertisements to promote smoking cessation. We conducted an RCT showing how 10 advertisements developed by a public health practitioner could influence the likelihood that Internet search engine users would subsequently conduct searches related to smoking cessation. We then asked whether some advertisements are more effective than others, and explored variation by demographic group.

## Methods

### Overview

We conducted an RCT using the Bing Ads system (Microsoft Corporation, Redmond, WA, USA) to randomly display 10 different advertisements, and then followed exposed or unexposed participants to explore whether they were more likely to subsequently search for ways to quit smoking. The control intervention was “usual care,” meaning users were served whatever advertisements the Bing Ads system would have otherwise served.

We asked a public health professional to design textual advertisements. These advertisements conformed to basic public health communications campaign theory. They were designed without the assistance of an advertising firm to better reflect real-life public health advertisements, which are usually not designed with such expertise. We then categorized the advertisements into 1 or more of 3 categories according to whether the text (1) empowered participants to quit (“empowering”), (2) suggested ways to quit (“helping”), or (3) discussed the effects of smoking on one’s health (“effects”). We then linked these advertisements to the most commonly visited smoking cessation websites (as determined by Microsoft’s search engine, Bing). These antismoking sites were operated by a government body, a nongovernmental organization, or a private entity, and thus were chosen because of the perceived difference in authority of each organization type.

### Advertisements

**Figure 1:**
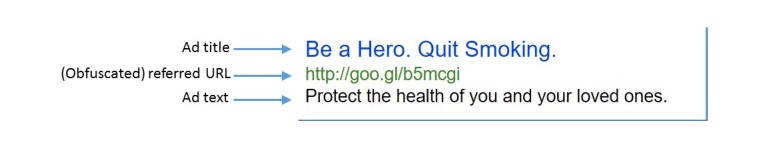
Sample advertisement promoting smoking cessation aimed at users of the Bing search engine.

The advertisements contained text and took the form of a title, a body, and a link to a URL (Table 1). We used a full factorial design such that we tested all combinations of title/body and URL. The advertisements were only shown to people who searched from computers located in the United States, and who performed searches on the Bing search engine (Microsoft Corporation). Figure 1 shows a sample advertisement. We note that the market share of Bing in the United States is around 19%, according to recent estimates [15]. The correlation between the number of Bing users per county in the United States and the number of people in that county according to the 2010 US Census is *R*^2^=.83 (*P*<.001). Thus, it is estimated that Bing users are a representative sample of the US population.

**Table 1:** Smoking cessation advertisement title and subtitle text and classification.

Title	Subtitle text	Classification
Stop It!	Smoking lowers quality of life and causes illness and death. Quit now.	Effects
Be a Hero. Quit Smoking.	Protect the health of you and your loved ones.	Empowering
Smoking Makes You Ugly	See the effects of smoking on your looks and health.	Effects
Quit Smoking Your Way	Find a quit method that will work for you!	Helping
Free Help to Quit Smoking!	Tips and tools to help you quit smoking. Choose what’s best for you!	Helping
Stop While You Still Can	Tobacco use is responsible for 1 in 5 deaths in the US. Quit smoking.	Effects
Who Says Quitting is Bad!	Learn about the immediate benefits of quitting smoking.	Helping
Want to Ruin Your Life?	Smoking harms nearly every organ of the body. Smoking also kills.	Effects
Quitting Smoking Is Hard	Having a plan to quit smoking makes it easier. Get help to prepare one.	Empowering, Helping
Need Motivation to Quit?	Learn from former smokers who have smoking-induced illness and disability.	Helping

**List of terms matched in users searches on “from smoking” on the Bing search engine.**

1. cancer from smoking

2. birth defects from smoking

3. yellow teeth from smoking

4. black lungs from smoking

5. hairy tongue from smoking

6. hole in throat from smoking

7. diseases from smoking

8. hole in neck from smoking

9. damaged lungs from smoking

10. wrinkles from smoking

The advertisements were shown when users’ searches were matched by (1) broad terms (eg, those searches containing the words “smoking” or “cigarettes”) or (2) specific terms (eg, “smoking causes black lungs”). These specific terms were identified by finding the 10 most common queries submitted during November 2014 to the Bing search engine that contained the phrase “from smoking.” Textbox 1 shows the list of terms.

The advertisements were randomly shown either directly underneath the search text (“top of the page”) or to the right of the search results (“right of the page”). The advertising system sometimes presented our advertisements in addition to other advertisements paid for by sponsors. In those cases, a placement at “location 1” meant that the advertisement was shown as the first advertisement in the list, and lower locations implied a lower order within the set of advertisements shown.

The Bing Ads system chose which of the 10 advertisements to show randomly using a random number generator. Each of the 10 advertisements had the same probability of being displayed. While it is likely that most users saw the advertisements, only some users actually clicked on it. Upon clicking, they were led to antismoking sites that we had preselected from government websites, nongovernmental organization websites, or commercial websites. We selected these websites according to their search rankings. Importantly, the sites did not contain detailed smoking cessation information. Therefore, we used future searches to test the effect of the advertisements on seeking cessation information, as detailed below.

The advertisements were shown between June 10, 2015 and September 10, 2015.

### Statistical Analysis

We extracted all searches conducted on Bing by people who were shown 1 or more of the advertisements we presented from 1 month before the beginning of the advertisement campaign (ie, May 10, 2015) and until 1 month after its completion (October 10, 2015). This was done to obtain a sense of the baseline number of searches and the advertisement types that these searches generated. This way, we could more easily detect a media event (eg, a celebrity death attributable to smoking) in the middle of a campaign and account for variations from baseline that could have occurred as a result.

Searches comprised the text of searches, date and time, and an anonymous user identifier. Where users had a Microsoft account and were signed in, their age and gender, as well as the zip code location of users, were also recorded.

We categorized searches according to their text into a 3-level scale: we assigned a score of 0 to searches that only mentioned “smoking” or “cigarettes,” without additional context. We gave a score of 1 to searches that mentioned symptoms associated with smoking, such as “wrinkles from smoking.” We gave searches a score of 2 if they mentioned specific diseases associated with smoking, such as “cancer from smoking.” We refer to this scale as the query damage scale.

Our primary outcome of interest was whether the user conducted at least one query, filtered to include only those queries indicative of an intention to quit smoking search (IQSS). Such queries were those that included 1 or more of the following terms: (1) direct reference: quit, cessation, stop smoking, smoking withdrawal; (2) smoking cessation medications: nicotine, bupropion, carenicline, Chantix, Buproban, Aplenzin, Wellbutrin, Budeprion, Zyban, clonidine, Catapres, Kapvay, Nexiclon, Clophelin, Nicoderm, Nicorette; (3) electronic cigarettes: e-cigarettes, electronic cigarette, vaporizer, vape, smokeless, vapor; and (4) support: support group, Smokefree, smoke free, American Cancer Society, American Lung Association.

We constructed a Cox proportional hazards model to assess the relation between the likelihood of conducting subsequent IQSS and the independent attributes shown in Textbox 2.

**Independent attributes.**

1. Attributes of the advertisements:

   a. Location on the page: either above the search results or to their right.

   b. Whether the advertisement was clicked on.

   c. Type of match between the query and the ad keywords: advertisements can be exact (the exact phrase used for triggering the advertisements is         the query) or approximate (the words triggering the advertisement appear in query, as well as other words).

   d. The websites they referred to (government, nongovernmental organization, or other).

2. The search words that triggered the advertisements.

3. User demographics:

   a. Age.

   b. Gender.

The assumption of proportionality was tested and met. To measure advertisement (and advertisement parameter) success, we further computed a conversion fraction, defined as the fraction of advertisement displays that resulted in a future IQSS. We then defined the conversion fraction ratio (CR) as the ratio of conversion fractions when an attribute was present to when it was not. Because the number of participants was very large, we only considered differences of 10% to be clinically meaningful irrespective of the statistical significance of the finding.

### Institutional Review Board Approval

Our study was approved by the Microsoft Institutional Review Board and was declared exempt by the Columbia University Institutional Review Board under the understanding that the Columbia University researchers would not have access to the data and would not seek funding for the study.

## Results

The advertisements were shown 710,106 times and clicked on 1167 times. Of these showings, 171,297 were linked to 3086 users, who also conducted 663,493 searches on Bing. Table 2 shows statistically significant advertisement and user attributes relative to the hazards of IQSS for those who were exposed to a smoking cessation advertisement of any type relative to those who were not exposed to an advertisement. Table 2 shows that advertisements placed on the top right of the page were twice as likely (hazard ratio [HR] 2.11, standard error [SE] 0.067) to induce subsequent searches indicating IQSS (a search for some kind of smoking cessation product or advice). Additionally, advertisements placed below those that did not have smoking cessation content were more effective than those placed at the top of a list (HR 1.13, SE 0.020 relative to the bottom of a list). Participants were only 1% more likely to subsequently conduct IQSS for each time that they were exposed to an advertisement (HR 1.01, SE 0.001). This suggests that 10 advertisements would be needed for a 10% increase in near-term smoking cessation-related searches.

Men were less likely than women to have future IQSS (HR 0.24, SE 0.073). Older participants who were exposed to smoking cessation advertisements were 2% more likely to subsequently search for IQSS for each additional year of the participant’s age than were those who were not exposed to such advertisements in a linear fashion (SE 0.002).

Advertisements that had empowering text, those that had text that suggested help, and those that discussed the effects of smoking tended to induce more IQSS. Websites produced by nongovernmental and commercial websites were more likely to induce IQSS than were governmental websites overall.

**Table 2:** Results of a Cox proportional hazards model with intention to quit smoking search terms as the dependent variable.^a^

Variable	Hazard ratio	SE^b^	*P* value
Ad shown on top of page (to the right)	2.11	0.067	<.001
Ad rank (top to bottom of list)	1.13	0.020	<.001
Number of previous exposures to ad	1.01	0.001	<.001
Empowering ad? (Not empowering)	2.57	0.092	<.001
Helping ad? (Not helping)	2.46	0.115	<.001
Ad discussing effects? (Not effects)	1.36	0.138	0.03
Government website (Not government)	0.55	0.106	<.001
NGO^c^ website (Not NGO)	0.63	0.094	<.001
Query damage scale	0.55	0.091	<.001
Gender (male)	0.24	0.073	<.001
Age (years)	1.02	0.002	<.001

^a^Hazards reflect exposure to the treatment (smoking cessation advertisement) relative to the control (no advertisement) by selected advertisement characteristics (reference characteristic). Only statistically significant variables are shown.

^b^SE: standard error.

^c^NGO: nongovernmental organization.

**Figure 2:**
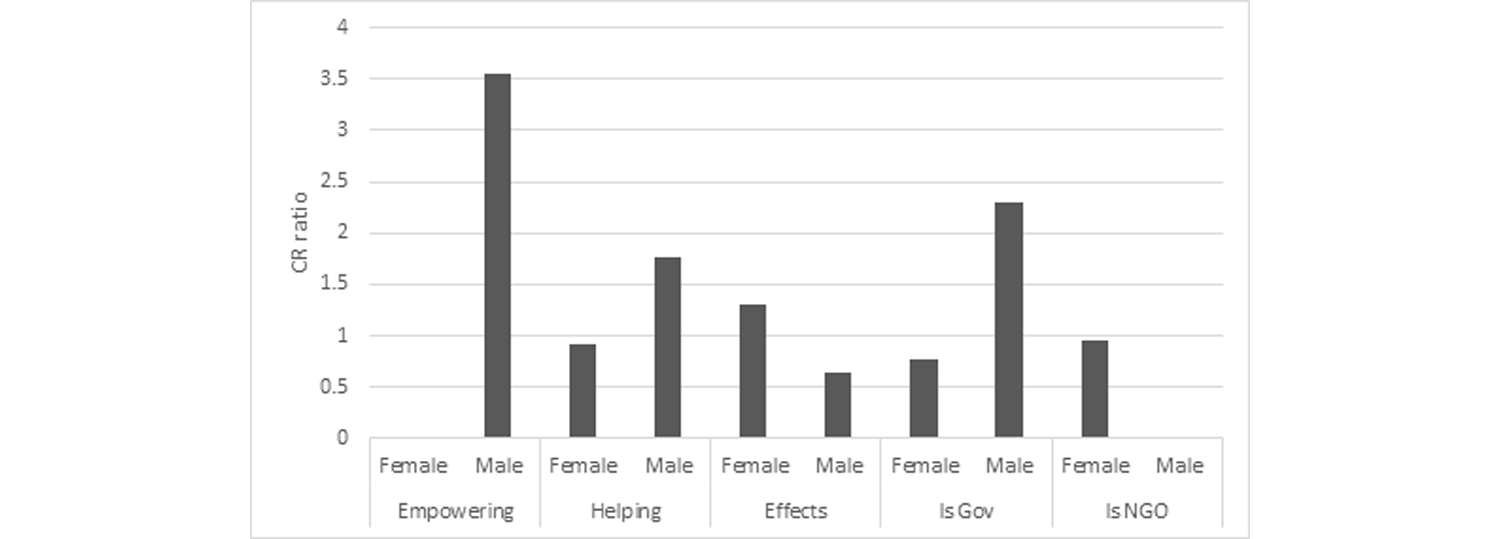
The conversion ratio (CR) during the first 2 days compared with all other times for advertisement attributes (“empowering” ads, ads “helping” participants to quit, and ads showing the “effects” of smoking), website attributes (governmental [gov] and nongovernmental organization [NGO]), and sex. A higher CR implies that more users shown the ad subsequently conducted a search related to smoking cessation. All effects are statistically significant at *P*<.05 (χ^2^_1_ test), except the following pairs: Helping-Female and Is NGO-Female.

Recall that the CR refers to the likelihood of IQSS when an attribute is present relative to when it is missing. A higher CR indicates that more users shown the advertisement subsequently conducted an IQSS. As Figure 2 shows, men were 3.5 times more likely to respond to empowering advertisements than to advertisements in other categories. Men were also more than twice as likely to respond to advertisements provided on government websites relative to other websites. Women were roughly 1.3 times more likely to respond to advertisements that stressed the effects of smoking relative to those that did not.

We further modeled the data to predict which users would likely perform smoking cessation searches in the 2 days following exposure to the advertisement, given their personal attributes, as well as those of their search and of the advertisements. To do this, we built a linear regression model with interactions, estimating its performance as measured by the area under the receiver operating characteristic curve (AUC) using 5-fold cross-validation [16]. The AUC of this classifier was 0.892.

After performing sequential forward feature selection [16], we found that by using 7 of the 13 attributes, we could obtain an AUC of 0.870. The 7 variables were advertisement shown on top, advertisement position, age, gender, empowering advertisement, URL referring to a government website, and the query damage scale.

Table 3 shows statistically significant (*P*<.05, with Bonferroni correction) attributes. The interactions in the model reveal that people of different ages responded differently to advertisement placement. Similarly, different advertising content affected people of different genders and ages in a dissimilar manner. Thus, tailoring the advertisements to specific audiences requires the selection of very specific advertisement wording, placements, and URLs.

**Table 3:** Statistically significant attributes and their interactions for predicting future smoking cessation searches.

Variable	Coefficient	SE^a^
Gender	–.0130	0.0027
Ad shown on top	.0208	0.0039
URL designed by government	.0383	0.0041
Age × ad position	.0001	0.00001
Age × empowering	.0009	0.0001
Age × ad shown on top	–.0010	0.00005
Age × government website	–.0008	0.0001
Ad position × damage scale	–.0023	0.0005
Ad position × empowering	.0089	0.0009
Ad position × ad shown on top	.0109	0.0008
Ad position × government website	–.0050	0.0006
Damage scale × ad shown on top	–.0104	0.0020
Empowering × gender	.0202	0.0027
Empowering × ad shown on top	.0172	0.0032
Empowering × URL is government	–.0994	0.0062

^a^SE: standard error.

## Discussion

In this study, we explored the effect of various advertisement characteristics on participants’ likelihood of conducting future searches related to smoking cessation. Our results show that men were more likely to subsequently conduct smoking cessation searches when exposed to advertisements containing empowering content, but women were more influenced by advertisements that emphasized the health effects of smoking. We also found that women and older people were generally more likely to be influenced by antismoking advertisements, and that placement of smoking cessation advertisements in the middle of a list of unrelated advertisements can improve their effectiveness in prompting future searches related to smoking cessation.

Thus, our results indicate a good deal of variation in the likelihood of future smoking cessation searches that is explained by the characteristics of the advertisements, the characteristics of the participants, and the type of entity that produced the website. Foremost, we found that smoking cessation advertisements that empowered individuals to quit were more effective among men, whereas those that suggested ways to quit were more effective among women. Further, we found that the landing page had implications for users’ likelihoods of subsequent smoking cessation searches.

Overall, our findings suggest that targeting Web-based advertising may improve the effectiveness of those advertisements. Therefore, it is possible that relatively simple alterations in ad-serving algorithms can improve public health. Likewise, the landing page can also differ depending on the characteristics of the user. Based on our results, we would want to rely more on government websites as landing pages when the searcher is young, for instance. That women are more susceptible to Web-based advertisements (irrespective of their placement or content) is encouraging, as this is the group for which mortality has been on an unprecedented rise due to smoking [17].

There were some important limitations to this work. Mainly, our study was limited by a lack of comprehensive data. First, it is unclear that subsequent searches actually translated into changes in smoking behavior—and without such data, our understanding of the health consequences of advertising is limited. Second, our demographic targeting was less specific than we would have liked. In the ideal, it would be possible to use information stored on users’ computers to devise Bayesian prediction algorithms. Such algorithms can be used to devise typologies for individual users (not just age and gender), and then to design much more targeted advertisements for such users.

However, there remains a clear challenge in understanding the influence of Web-based advertising on public health. Although private corporations conduct tens of thousands of such experiments each year, academics are required to obtain the consent of all participants in human subjects research. Even with informed consent, the privacy challenges associated with working with potentially identifiable data are daunting. For these reasons, academics who would be naturally positioned to better understand the public health consequences of Web-based advertising targeting cannot directly engage in such research. In this way, institutional review standards that have not pivoted to accommodate the volume and availability of data in the Internet age limit the role of those trained and experienced in public health research.

Our findings—that the characteristics of the advertisement’s content, the placement of the advertisements, and the entity producing the advertisement should vary by the demographic characteristics of the searcher—are an important first step in demonstrating the power of Web-based advertising. Future research should seek to refine advertisements based on demographic data, as well as to improve their design and delivery with the support of advertising professionals. Ultimately, this work has the potential to improve the effectiveness and efficiency of public health advertising to promote healthy behavior and mitigate chronic disease.

## Abbreviations

AUC: area under the receiver operating characteristic curve
CR: conversion fraction ratio
HR: hazard ratio
IQSS: intention to quit smoking search
RCT: randomized controlled trial
SE: standard error
